# Experimental Control of Macrophage Pro-Inflammatory Dynamics Using Predictive Models

**DOI:** 10.3389/fbioe.2020.00666

**Published:** 2020-07-13

**Authors:** Laura D. Weinstock, James E. Forsmo, Alexis Wilkinson, Jun Ueda, Levi B. Wood

**Affiliations:** ^1^Parker H. Petit Institute for Bioengineering & Bioscience, Georgia Institute of Technology, Atlanta, GA, United States; ^2^The Wallace H. Coulter Department of Biomedical Engineering, Georgia Institute of Technology, Atlanta, GA, United States; ^3^School of Chemical & Biomolecular Engineering, Georgia Institute of Technology, Atlanta, GA, United States; ^4^George W. Woodruff School of Mechanical Engineering, Georgia Institute of Technology, Atlanta, GA, United States

**Keywords:** macrophages, dynamic systems and control, inflammation, trajectory planning, system identification, predictive model

## Abstract

Macrophage activity is a major component of the healthy response to infection and injury that consists of tightly regulated early pro-inflammatory activation followed by anti-inflammatory and regenerative activity. In numerous diseases, however, macrophage polarization becomes dysregulated and can not only impair recovery, but can promote further injury and pathogenesis, e.g., after trauma or in diabetic ulcers. Dysregulated macrophages may either fail to polarize or become chronically polarized, resulting in increased production of cytotoxic factors, diminished capacity to clear pathogens, or failure to promote tissue regeneration. In these cases, a method of predicting and dynamically controlling macrophage polarization will enable a new strategy for treating diverse inflammatory diseases. In this work, we developed a model-predictive control framework to temporally regulate macrophage polarization. Using RAW 264.7 macrophages as a model system, we enabled temporal control by identifying transfer function models relating the polarization marker iNOS to exogenous pro- and anti-inflammatory stimuli. These stimuli-to-iNOS response models were identified using linear autoregressive with exogenous input terms (ARX) equations and were coupled with non-linear elements to account for experimentally identified supra-additive and hysteretic effects. Using this model architecture, we were able to reproduce experimentally observed temporal iNOS dynamics induced by lipopolysaccharides (LPS) and interferon gamma (IFN-γ). Moreover, the identified model enabled the design of time-varying input trajectories to experimentally sustain the duration and magnitude of iNOS expression. By designing transfer function models with the intent to predict cell behavior, we were able to predict and experimentally obtain temporal regulation of iNOS expression using LPS and IFN-γ from both naïve and non-naïve initial states. Moreover, our data driven models revealed decaying magnitude of iNOS response to LPS stimulation over time that could be recovered using combined treatment with both LPS and IFN-γ. Given the importance of dynamic tissue macrophage polarization and overall inflammatory regulation to a broad number of diseases, the temporal control methodology presented here will have numerous applications for regulating immune activity dynamics in chronic inflammatory diseases.

## Introduction

**Figure 1:**
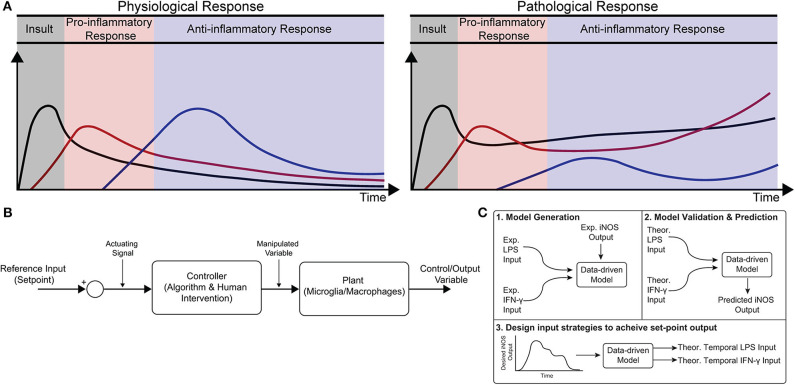
Conceptual diagram of modeling immune response in health and disease. **(A)** Immune response as dynamically regulated in health (left) and dysfunctional in chronic conditions (right). **(B)** Block diagram with macrophages as the “system” or “plant” that is being controlled. **(C)** Identification, validation, and prediction of inflammatory response as a three-step process consisting of (1) design of an engineering model structure and fit of model parameters, (2) comparison of predicted and experimental results, and (3) use of the predictive model to design input strategies to obtain a desired response.

Healthy immune response during infection or injury is a dynamic process consisting of initial acute pro-inflammatory activation followed by anti-inflammatory/resolving activity, which is mediated in large part by macrophages (Sica and Mantovani, 2012; Decano and Aikawa, 2018). This temporally regulated response promotes pathogen and debris clearance followed by tissue regeneration and, ultimately, recovery of homeostasis (Figure 1A; Sica and Mantovani, 2012; Decano and Aikawa, 2018). Dysregulation can occur in several ways. First, a strong initial pro-inflammatory response within the affected tissue can lead to systemic inflammation that positively feeds back to sustain local inflammation. Second, a compensatory anti-inflammatory response (e.g., via regulatory T cells) can lead to aberrant immunosuppression, which impairs pathogen clearance and regeneration (Binkowska et al., 2015). Third, long-term dysregulation of immune response during chronic disease interferes with tissue regeneration and homeostasis, in turn further sustaining immune dysregulation. Indeed, chronic inflammatory dysfunction contributes to a breadth of diseases, including impaired wound healing after major trauma and multiple neurodegenerative diseases (Figure 1A; Ohashi et al., 2015; Oishi and Manabe, 2016), and chronically impaired immune response can lead to worsened outcomes after new insults (Wynn et al., 2013). However, broad ablation of immune response, e.g., via corticosteroids, can equally limit successful regeneration, and recovery of tissue homeostasis (Guo and Dipietro, 2010; Weekman et al., 2014; Oishi and Manabe, 2016; Hamelin et al., 2018).

Although the need for regulation of tissue immune response is well-recognized, identification of new strategies to intervene in tissue inflammation remains a major challenge. After trauma for example, treatment selection, dosing, and timing of administration are all crucial factors in determining patient outcome (Becelli et al., 2000). There has recently been a call for a better understanding of the complex and dynamic immune response post-injury in order to identify new strategies to regulate dynamic immune response and ultimately patient outcome (Galbraith et al., 2016).

The dynamic activity of macrophages is integral to both the early (<1 h) and continued (>1 month) response to infection and injury (Wynn et al., 2013; Hu et al., 2015). Without appropriate regulation of their activity, macrophages can drive the initiation and progression of many diseases (Wynn et al., 2013; Ohashi et al., 2015). In particular, loss of regulation can lead to insufficient pro-inflammatory activity, leading to incomplete clearance of pathogens and/or tissue debris, impaired pro-regenerative response, chronic inflammation, and infection (Guo and Dipietro, 2010; Oishi and Manabe, 2016). Recent efforts to regulate dysfunctional macrophages have focused on cell-based therapies, such as delivery of mesenchymal stem cells (MSCs) or macrophages conditioned *ex vivo* toward anti-inflammatory and pro-regenerative “M2” phenotypes. The underlying principal behind immunomodulatory cell therapies is that these cells will act as natural “controllers” of immune response through beneficial immunomodulatory signaling in the local environment (Pacini, 2014). However, these strategies are subject to a number of limitations. For example, MSCs are subject to variable efficacy between donors and batches (Wang et al., 2012; Pacini, 2014). Other approaches seek to deliver *ex vivo* modified macrophages, but both mouse and human trials have had variable success and still face many challenges (Lee et al., 2016; Spiller and Koh, 2017). A new approach that actively regulates resident tissue macrophages would escape many challenges faced by current cell-based therapies.

Exogenous control of macrophage activity would provide an exciting new method to modulate immune response (Ohashi et al., 2015; Decano and Aikawa, 2018) that would steer the system through a desired trajectory of activity. Macrophages are an attractive target for regulating immune response because (i) they are involved in diverse immune functions essential for tissue protection and repair and (ii) they are highly plastic, with the ability to dynamically re-polarize for different functions based on external cues (Wynn et al., 2013). Since macrophage polarization is dynamic, a quantitative temporal model will enable design of exogenous input sequences capable of normalizing response (Figures 1A,B). The pathways governing macrophage polarization in response to stimuli have been comprehensively modeled, including receptor binding kinetics, downstream kinase signaling, and gene transcription (Salim et al., 2016). While mechanistically appealing, these models possess dozens of equations and hundreds of parameters, making it intractable to identify reliably predictive input-output relationships between exogenous stimulation and polarization in terms of these precise mechanistic models. Moreover, it has recently been argued that identification of viable strategies to intervene in immune activity will require rigorous integration of experimental data with computational modeling (Vodovotz et al., 2017). There is thus a need for an empirical input/output model that relates macrophage response to exogenous inputs in order to predict and control activation levels over time.

In the current study, we formulated a data-driven modeling approach, informed by an *in vitro* macrophage polarization assay and system identification theory, to identify the temporal dynamics of macrophage response to multiple exogenous pro-inflammatory stimuli. Specifically, we conditioned RAW 264.7 macrophages with M1 polarizing stimuli (LPS and IFN-γ) or an M2 polarizing stimulus (IL-4) and quantified response in terms of iNOS expression for 1–72 h post-stimulation. We then used least squares regression to fit a low-order autoregressive with exogenous terms (ARX) model together with non-linear elements to relate iNOS response to each input (Figures 1C1,2). The identified model predicted the dynamics of polarization in subsequent experiments in response to different concentrations and temporal trajectories (simultaneous vs. sequential) of each input (Figure 1C3). Finally, we used the identified model as part of an open-loop control framework to tailor input sequences to achieve desired temporal trajectories of macrophage polarization *in vitro*. To our knowledge, this is the first study to experimentally control immune cell dynamics using a predictive control framework. Given the importance of dynamic M1 and M2 polarization during tissue regeneration, the control methodology presented here defines a novel framework that will have diverse applications for treating chronic inflammatory diseases and promoting tissue regeneration.

**Figure 2:**
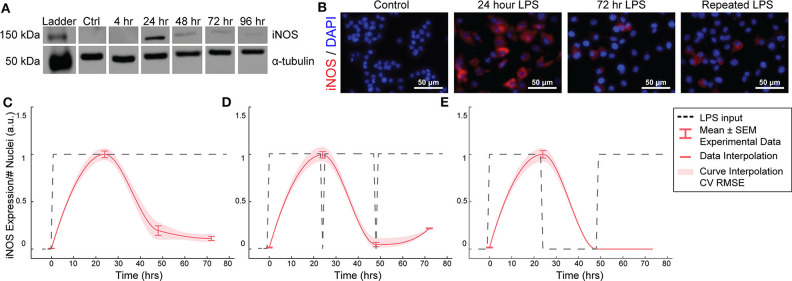
RAW264.7 macrophages transiently express iNOS in response to constant or repeated LPS stimulation. **(A)** Representative Western blot for iNOS (140 kDa) and α-tubulin (55 kDa) after LPS treatment. **(B)** Representative ICC images showing iNOS response after LPS stimulation. **(C)** ICC quantification matches Western blot analysis of transient iNOS expression in response to a single administration of LPS. **(D)** Dynamics of iNOS expression are not modulated in response to multiple administrations of LPS or **(E)** after 24 h in basal medium before LPS re-stimulation (mean ± SEM, *N* = 16 at 0, 24, 48, and 72 h; red curves; interpolation ± RMS CV error).

## Results

### Macrophage iNOS Expression Is Transient and Refractory to Repeated Stimulations

We first aimed to determine the temporal dynamics of macrophage response to single or repeated pro-inflammatory stimuli. As a model system, we used expression of the pro-inflammatory M1 marker inducible nitric oxide synthase (iNOS) by RAW 264.7 macrophages in response to the pro-inflammatory stimulus lipopolysaccharide (LPS). Using quantitative Western blot, we found that a single administration of 1 μg/mL LPS, but not IL-4 (Supplementary Figure S1), resulted in transient iNOS dynamics with a peak in iNOS expression at 24 h followed by a decay to baseline over the following 48 h (Figure 2A). Immunocytochemistry (ICC) confirmed this response (Figures 2B,C) and revealed that this temporal trajectory was (1) conserved given a range of lower doses of LPS and (2) that the magnitude of the response monotonically increased with the magnitude of the stimulation (Supplementary Figure S2). Intriguingly, although LPS was not removed from cultures, and thus represented a persistent step-like stimulus, the dynamics of iNOS expression followed a first order decay response (Figures 2B,C). In traditional engineered systems, this type of system response is usually obtained by stimulating the system with a finite impulse input (Ljung, 1999).

To test whether the observed decay in iNOS expression was due to LPS depletion from the culture medium, we re-administered 1 μg/mL LPS every 24 h. However, iNOS expression in response to repeated stimulation was comparable to that of a single LPS stimulation (Figure 2D), indicating suppression of response to continued stimulation, which is consistent with known auto-inhibitory mechanisms of macrophage response to LPS, such as induction of ATF3 (Lawrence and Natoli, 2011) and kinase phosphatases (Zhao et al., 2006; Sun et al., 2017). Although the dynamics of these auto-inhibitory processes have not been fully delineated, we next wanted to determine if we could identify a stimulation strategy that would increase sustained iNOS expression over the course of our 72 h culture experiments. Because we found an initial peak at 24 h in response to 1 μg/ml of LPS, we tested a recovery time period of 24 h between the initial peak and a potential second peak within the 72 h experimental treatment window. However, cycled re-stimulation did not alter iNOS expression dynamics (Figure 2E), suggesting that the dynamics of macrophage polarization to LPS stimulation consist of an initial response that is not sustained despite either continued or repeated LPS stimulation, during our experimental time window, i.e., the system becomes refractory. This refractory behavior resembles immune tolerance/fatigue observed in chronic disease conditions, such as type 2 diabetes and cancer (Geerlings and Hoepelman, 1999; Makkouk and Weiner, 2015).

### Auto-Regressive Model With Exogenous Inputs Fits iNOS Dynamic Response to LPS Input

We next asked if a control systems engineering methodology could be used to design a temporal sequence of LPS stimulation that would enable us to recover or sustain iNOS expression, and, by extension, pro-inflammatory activation of RAW 264.7 cells. Control systems methodology requires a model that can be used to predict future system response given a known stimulation input. Diverse model structures are employed in engineering fields, ranging from high-order mechanistic models to input-output data-driven models. For this application, a mechanistic model encoding all of the genetic and protein interactions responsible for iNOS expression would suffer from reduced predictive capacity due to uncertainty in fitted parameters. Gray and black box models, which capture dominant response dynamics without specifying mechanistic details, are thus more appealing to relate iNOS dynamics to pro-inflammatory stimulation (Shin et al., 2012). We therefore sought to identify an optimized black box single input and single output (SISO) model relating LPS input to iNOS output (Shin et al., 2012; Rachad et al., 2015). A critical tradeoff must be considered when choosing model structure: maximize flexibility to best capture system dynamics while avoiding the need to have more model parameters than can be reliably identified from the data (Van den Hof et al., 1994). Autoregressive models with exogenous inputs (ARX) models are frequently used for black-box system identification because they can capture underlying system dynamics in diverse applications and because parameterization using the ARX (Materials and Methods, Equations 1–3) structure guarantees uniqueness of solution and identification of the global minimum of the error function (Liu and Allen, 2002; Zurakowski and Teel, 2006; Shin et al., 2012; Deshpande et al., 2014).

**Figure 3:**
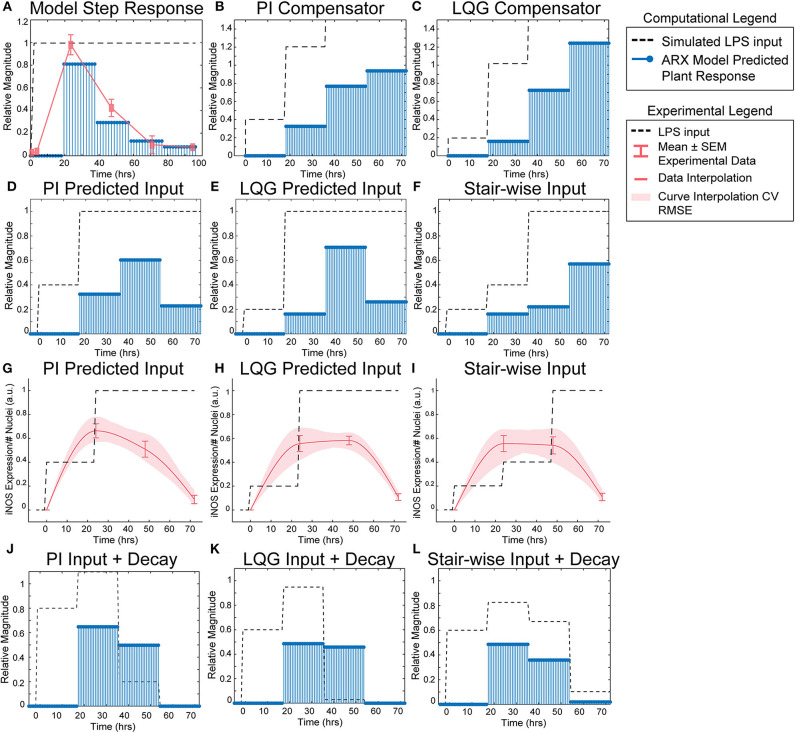
SISO LPS/iNOS ARX model, controller design, and experimental MPC testing. **(A)** Identified ARX model of macrophage iNOS response to LPS has a characteristic step response that follows the experimentally quantified trajectory. Control system design identifies input strategy (dashed line) for a step reference that elicits a gradual increase in plant response (blue stems) using a **(B)** PI or **(C)** LQG controller. Model simulations given controller defined inputs but within experimental input constraints predict sustained outputs for **(D)** PI and **(E)** LQG controllers. **(F)** A heuristically defined three-step increase input strategy predicts an output that reaches a maximum at 72 h. Experimental implementation using cultured RAW 264.7 macrophages and **(G)** PI controller-, **(H)** LQG controller-, or **(I)** a heuristic combination of designed LPS input schema (dashed line) modulates temporal iNOS expression (red curves, mean ± SEM, *N* = 16; interpolated curve ± RMS CV error) but does not reach the unit reference nor sustain 72 h activity. Macrophage refractory response to repeated LPS input is captured (blue stems) by multiplying the **(J)** PI predicted, **(K)** LQG predicted, or **(L)** heuristically defined input sequences against a time-dependent exponential decay term (dashed lines).

To identify the parameters of this model architecture, extensive experimental characterization of macrophage polarization dynamics with multiple input patterns and magnitudes was performed to generate a rich dataset to train and identify an input/output model of iNOS expression dynamics (Figures 2C–E, Supplementary Figure S2). We experimentally found that macrophages exhibited a monotonic LPS dose-to-iNOS response relationship within a physiologically relevant concentration range (Supplementary Figure S2), which is well-described using the linear ARX model structure. Above a high (1 μg/mL) concentration of LPS, response tapers off, potentially due to cell death or changes in intracellular signaling activity (Ziegler-Heitbrock et al., 1994). As such, we set 1 μg/mL LPS as the maximum concentration used in this study. To capture the post-LPS stimulation refractory period, we fit an ARX model (orders n_a_ = 1, n_b_ = 2, n_k_ = 1, Materials and Methods, Equations 1–3) to experimental time sequence input-output data from numerous experimental runs consisting of constant high input (*N* = 38), constant input for three lower concentrations (10, 100, and 500 ng/mL, *N* = 4), cyclic high input (Figure 2E, *N* = 8), and replenished high input (Figure 2D, *N* = 8) with model parameters estimated using least squares (Materials and Methods, Equation 4). The resulting model recapitulated this refractory pattern for a step input (Figure 3A). The model parameter estimates are given in Supplementary Table S1 (three free coefficients) and returned a normalized Akaike's Information Criterion (AICc) model quality metric of 430.59 and minimized mean squared error (Supplementary Table S3). This model outperforms the related ARMAX (autoregressive-moving average with exogenous terms) model structure with similar numbers of parameters (n_a_ = 1, n_b_ = 2, number of moving average coefficients n_c_ = 0; AICc = 501.96). By estimating this input/output model (Supplementary Tables S1, S2), we can achieve both high descriptive and predictive capacities.

### Model Predictive Controller Identifies LPS Stimulation Sequence to Sustain iNOS Expression

Using the identified ARX system model, we sought to tune a controller (Control System Design Toolbox, MATLAB), placed upstream of the plant (Figure 1B), that would predict a temporally defined LPS input strategy to overcome the persistent decay in iNOS expression. We used two controller structures to design input strategies capable of achieving sustained iNOS expression. First, since our system dynamics (Figure 2C) indicated that the system model responds to the derivative of the input, we attempted to compensate for the derivative using a classical proportional-integral (PI) controller, which is commonly applied in engineering applications to minimize steady-state error (Nise, 2015; Supplementary Table S4). Here, we used the PI controller (Materials and Methods, Equation 8) to control LPS-induced iNOS expression to the unit reference (1 a.u. iNOS relative expression, Materials and Methods). The controller predicted that a stair-wise delivery of LPS (Figure 3B, dashed line) would give rise to a more gradual but prolonged output response, *y*, that reached the reference by the control horizon of 72 h (Figure 3B, blue stems). Importantly, the second step in input exceeded the unit input value (corresponding *in vitro* to 1 μg/mL LPS), which was the upper bound of LPS concentration used in this study. When the controller was constrained to inputs between 0 and 1 (1 μg/mL LPS), no PI controller obtained by adjusting controller gains K_p_ and K_i_ (Materials and Methods), was capable of defining an input sequence that both maintained a *u* ≤ 1 μg/mL and predicted *y* to reach the reference within the control time horizon.

Due to the inability of the PI controller to identify an input sequence capable of reaching or maintaining output levels at 72 h, we next decided to take advantage of our ARX system model to re-design the input sequence using a linear-quadratic Gaussian (LQG) controller (Materials and Methods, Equation 9; Supplementary Table S4), which can provide improved performance over conventional PID controllers for minimizing total error (Mohammadbagheri et al., 2011). This LQG controller designed a reduced magnitude for the original input followed by the unit max of LPS input (Figure 3C, dashed line**)** to achieve 80% of the reference point prior to exceeding the unit max stimulation input (Figure 3C, blue stems), which the PI controller-defined input could not achieve within LPS concentration constraints. However, this controller also required *u*>1 μg/mL to reach the reference. When the input is constrained to 0 ≤ *u* ≤ 1 μg/mL LPS, the model simulations predicted that progressive step increases in LPS would prolong the iNOS response but not sustain it at the unit reference value (Figures 3D,E). Finally, when the initial magnitudes of the LQG and PI predicted inputs were heuristically combined in a three-step increase strategy, simulations predicted a maximum response at 72 h (Figure 3F).

### Experimental Implementation of Predicted LPS Input Temporarily Sustains Macrophage iNOS Activation

Each controller above defined a temporally increasing magnitude of the stimulus *u*, or LPS concentration, where the input is increased at each time step. Experimentally, the model predicted input values represent a fraction of the normalized maximum (high) LPS concentration, 1 μg/mL. For example, 0.2 is 20% of the maximum 1 μg/mL, or 200 ng/mL, and 0.4 is 400 ng/mL as in our data used for model fitting. To test the PI controller input strategy, RAW 264.7 macrophages were treated with 40 ng/mL of LPS for 24 h, followed by 1 μg/mL from hour 24 until fixation at 72 h (Figure 3G, dashed line**)**. Despite the controller requiring *u* of 1.2, biologically this would have led to excessive cell death, likely changing the plant response. Thus, we tested the effect of the unit max of LPS in this stair-wise input scheme. The macrophage expression of iNOS peaked at approximately 70% of normalized maximum iNOS (defined by the 24 h expression level given 1 μg/mL LPS) at 24 h (Figure 3G, red curve). The subsequent increase in LPS concentration delivered did not sustain this level of iNOS, which declines through the 48 and 72 h time points, but does keep levels higher (~50% max) at 48 h than an initially high level of LPS (Figure 3G, red curve).

The LQG controller predicted input, 24 h of 200 ng/mL followed by 48 h at 1 μg/mL LPS (Figure 3H, dashed line), realized an iNOS expression level ~60% of the reference at 24 h (Figure 3H, red curve). Intriguingly, here the cells sustained this iNOS level through 48 h, but not through 72 h (Figure 3H, red curve). We next heuristically combined the input strategies defined by the PI and LQG controller to test whether iNOS expression at 72 h could be sustained (Figure 3I, dashed line). However, iNOS expression given this strategy reflected that of the LQG controller and did not keep activation high at 72 h (Figure 3I, red curve).

The refractory, or muted, iNOS response to either high, continued, or step-wise increases in LPS stimulation suggested a decaying efficacy of LPS regardless of input sequence. Reduced response to LPS is consistent with time-dependent compensatory downstream signaling (Kadelka et al., 2019), including increases in phosphatases that down-regulate LPS-induced phospho-protein signaling, e.g., MAP kinase phosphatase 1 and Protein phosphatase 2A; inhibition of pro-inflammatory transcription factors; or up-regulation of anti-inflammatory transcription factors, e.g., STAT6 inhibition of NF-κB (Zhao et al., 2006; Lawrence and Natoli, 2011; Ni et al., 2016; Sun et al., 2017).

Because prior work has shown that signaling proteins downstream of LPS respond with exponentially decaying dynamics (Kadelka et al., 2019), we next hypothesized that an exponential decay term would improve agreement between our dynamic model and experimental data. Indeed, when the input sequence terms were multiplied by a time-dependent exponential decay term (Figures 3J–L, dashed lines), the response magnitudes (Figures 3J–L, blue stems) reflected the experimentally obtained iNOS values for each input strategy. Although this single input system was unable to meet constant reference control specifications, the ability to qualitatively maintain elevated pro-inflammatory macrophage activation via our predictive control framework demonstrated an exciting feasibility of the approach that may be extendable to alternate strategies that can overcome the decaying efficacy of LPS stimulation.

### IFN-γ Stimulation Increases Reachable iNOS Trajectories and Adds System Non-linearity

**Figure 4:**
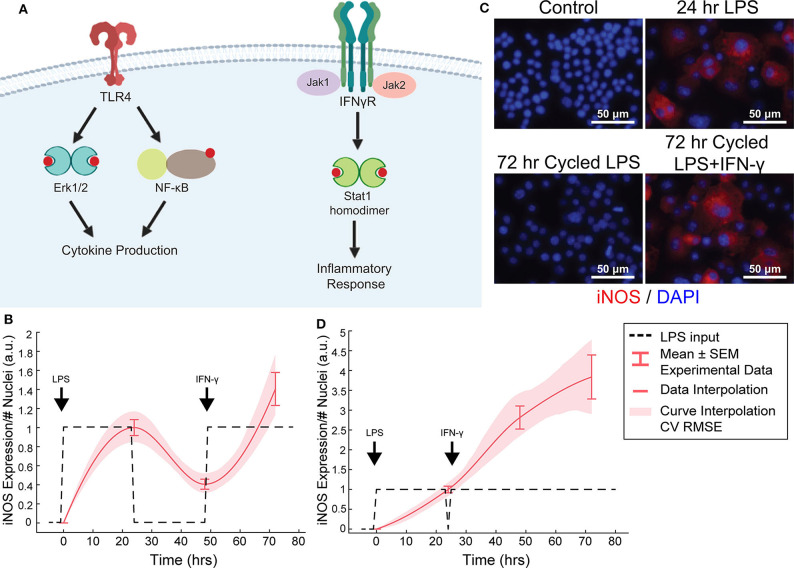
Orthogonal stimuli maintained or magnified iNOS expression. **(A)** Signaling diagram for LPS and IFN-γ (created with BioRender). **(B)** 24 h of LPS treatment and delayed subsequent IFN-γ (dashed lines) treatment modulates iNOS expression (red curves, mean ± SEM, *N* = 16; interpolated curve ± RMS CV error), even at 72 h time point. **(C)** Representative ICC images showing cycled LPS and IFN-γ (input defined in **B**) induces iNOS expression comparable to 24 h of LPS alone while cycling only LPS in that same pattern (Figure 2F) does not maintain expression. **(D)** 24 h of LPS treatment and immediately subsequent IFN-γ (dashed lines) treatment modulates iNOS expression (red curves, mean ± SEM, *N* = 16; interpolated curve ± RMS CV error), even at 72 h time point.

We found above that single or repeated stimulation with LPS was unable to indefinitely sustain iNOS expression and that sustained expression was only partially recovered by temporally modulating the input (Figures 3D–I), i.e., inflammatory activity was modulated but could not be prolonged indefinitely. In engineering systems, independent inputs increase the system rank and thereby increase state achievability. That is to say, adding a secondary stimulus that operates through separate, orthogonal means, expands the internal states, and reachable output of a system (Hespanha, 2009). Therefore, we next hypothesized that a second pro-inflammatory input would improve controllability. To test this, we used IFN-γ, which signals largely independently of LPS (Figure 4A) as the second, orthogonal input because 100 ng/mL IFN-γ robustly increased iNOS levels despite prior LPS input (Figures 4B–D). Although we also considered TNF-α as the second pro-inflammatory stimulus, we found the iNOS response is more sensitive to IFN-γ within a physiologically relevant concentration range (Supplementary Figure S3). Given these findings, the use of multiple pro-inflammatory inputs is promising for toggling both the magnitude and duration of macrophage activity with greater reachability than can be achieved with a single input.

**Figure 5:**
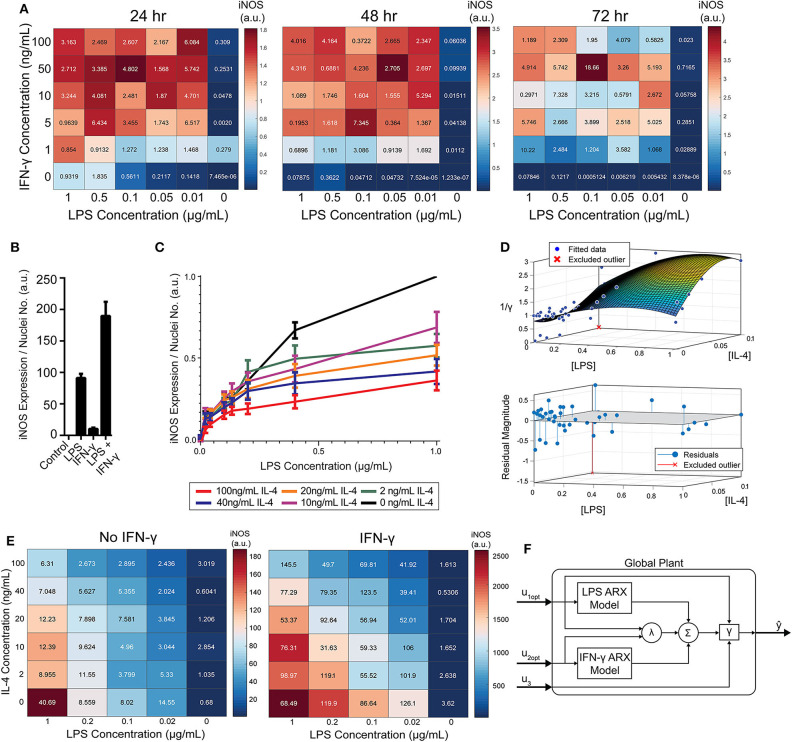
RAW 264.7 macrophages are markedly affected by activation state-dependent hysteresis, which can be overcome using multiple pro-inflammatory inputs. **(A)** LPS and IFN-γ added simultaneously cause time dependent supra-additive expression of iNOS (color represents mean, SEM displayed numerically, *N* = 2). Data are normalized by 1 μg/mL LPS-only condition for each time-point. **(B)** Selected non-normalized data from **A** (24 h, highest concentration per stimulus) demonstrating that iNOS expression from combined conditions is greater than the linear addition of LPS or IFN-γ alone (mean ± SEM, *N* = 2). **(C)** Prior treatment with IL-4 attenuates LPS induced iNOS expression (24 h post-LPS treatment) in an IL-4 concentration-dependent manner (mean ± SEM, *N* = 6). **(D)** Interpolated attenuation factor gamma surface plot (top) and fit error (bottom). **(E)** Pretreating macrophages with 100 ng/mL IL-4 for 24 h prior to LPS stimulation reduced the magnitude of pro-inflammatory polarization measured by iNOS expression normalized to DAPI (color represents mean, SEM displayed numerically, *N* = 4). Combining 4 ng/mL of IFN-γ with LPS stimulates iNOS expression, overcoming the hysteretic effect dependent on the dose of LPS (color represents mean, SEM displayed numerically, *N* = 4). **(F)** Diagram of global plant, as implemented in control system (Figure 1B), of multiple input system with both linear and non-linear model elements. System predicted inputs u_1_ (LPS) and u_2_ (IFN-γ) are fed into respective identified SISO ARX models and supra-additive interaction term λ elements. Terms multiplied by weighting coefficients c (defined by multiple regression estimation; Equation 10) prior to summation (Σ) and hysteresis-dependent attenuation (γ). Note that u_3_ accounts for IL-4 attenuation via γ.

While IFN-γ recovered iNOS expression from LPS-induced tolerance, it also introduced a non-linear element to the dynamic response—supra-additivity. ARX and transfer function models require that the output of the sum of two inputs equal the sum of the output of each input. However, IFN-γ amplifies LPS-induced iNOS expression, where expression is greater than the sum of expression from each stimulus alone, whether added concomitantly or in series. In fact, supra-additivity for simultaneous conditioning is present across all time points and for a range of LPS and IFN-γ concentrations through 72 h of conditioning (Figures 5A,B, Supplementary Figure S4). The supra-additivity also lead to iNOS expression that was greater than the unit reference for 24 h of LPS (Figures 5A,B), so our predictive model needs to account for these non-linearities to avoid overshooting or behavior that does not settled to the desired reference (Figure 4D).

### RAW 264.7 Macrophages Exhibit State Memory Based on Stimulation History

In disease, macrophages may exist in chronically activated or other non-naïve states, driven by local and systemic changes in signaling proteins, hormones, among other factors (Mosser and Edwards, 2008; Ohashi et al., 2015). Thus, having shown our ability to model macrophage pro-inflammatory dynamics and design input trajectories for naïve macrophages, we next wanted to determine whether the macrophage response to pro-inflammatory stimulation would be affected by pre-polarizing the cells toward an anti-inflammatory state.

To model RAW 264.7 cells starting in a non-naïve state, we pre-conditioned macrophages with IL-4 for 24 h prior to pro-inflammatory stimulation. Upon stimulation with LPS, we found that prior IL-4 conditioning attenuated expression of iNOS after 24 h of treatment with LPS, but that iNOS still responded to LPS in a concentration dependent manner (Figure 5C). M2 polarization was validated by increased expression of Arg1 (data not shown). Further, an initial polarization toward a pro-inflammatory phenotype increased the magnitude of anti-inflammatory polarization that outweighed the IL-4 concentration given (Supplementary Figure S5), which is consistent with prior studies, including one study where AAV delivery of IFN-γ *in vivo* increased M2 gene expression, as well as M1 genes (Weekman et al., 2014). Together, these data suggest that macrophages exhibit hysteresis in their response to prior inputs, whereby prior M2 polarization attenuates future M1 response and prior M1 polarization sensitizes future M2 response. The M2 driven attenuation of M1 response reflects one aspect of how systemic immunosuppression poses a major risk to post-traumatic or surgical injury patients (Kimura et al., 2010; Islam et al., 2016).

### Modeling Multi-Input Driven Hysteresis and Supra-Additivity

Since the dynamics of iNOS expression in RAW 264.7 cells were dependent on the polarization state history (i.e., hysteresis in non-naïve cells) and demonstrated supra-additivity in response to combinations of LPS and IFN-γ, we next sought to incorporate these elements into our iNOS response model. In terms of state history, quantification, and mathematical modeling of state-history dependence has previously been reported for cancer cell epithelial-mesenchymal transition (Celia-Terrassa et al., 2018; Tripathi et al., 2020). Here, we accounted for the hysteretic effects of prior treatment with IL-4 by defining an attenuation factor to account for the reduction in magnitude of iNOS expression in the next time step for the range of LPS and IL-4 concentrations described in Figure 5C relative to expression with no exposure to IL-4. Quantitatively, the attenuation factor γ (Materials and Methods) is equal to 1 for non-hysteretic systems and increases with higher prior concentrations of IL-4 such that $\frac{1}{\gamma }$ multiplied by iNOS expression for a given LPS concentration gives the iNOS response for that LPS concentration and an IL-4 pre-treatment concentration. A response plane for γ was fitted with 3rd order polynomials in [LPS] and [IL-4] to define a smoothed continuous response surface from which any attenuation due to anti-inflammatory induction is returned (Figure 5D).

To account for supra-additive effects of multiple pro-inflammatory inputs, as done for the hysteretic surface, we populated time-dependent interaction term (λ) surface curves for the defined ranges of co-addition of LPS and IFN-γ. Excitingly, the supra-additivity of IFN-γ with LPS demonstrated the ability to recover the attenuation effect induced by IL-4. Indeed, greater iNOS expression was observed across lower LPS concentrations and higher IL-4 concentrations when IFN-γ co-stimulation was used compared with LPS stimulation alone (Figure 5E, note that the scale of response is an order of magnitude greater in the heat map with IFN-γ). This interaction effect motivates the need for a system plant model that processes both M2 and M1 inputs.

The global plant model was constructed and is described schematically in Figure 5F. The system receives the concentration of LPS (u_1_) and IFN-γ (u_2_) which are passed into their respective identified ARX models (Supplementary Table S2), the supra-additivity of LPS and IFN-γ was accounted for using λ, the pro-inflammatory contributions are summed and applied as inputs to the hysteresis term γ, Finally, the output is the predicted iNOS output (ŷ) as a function of time *t* (Figure 5F).

### Design of LPS and IFN-γ Temporal Input Trajectories With Global Plant Model Achieves Sustained iNOS Expression

**Figure 6:**
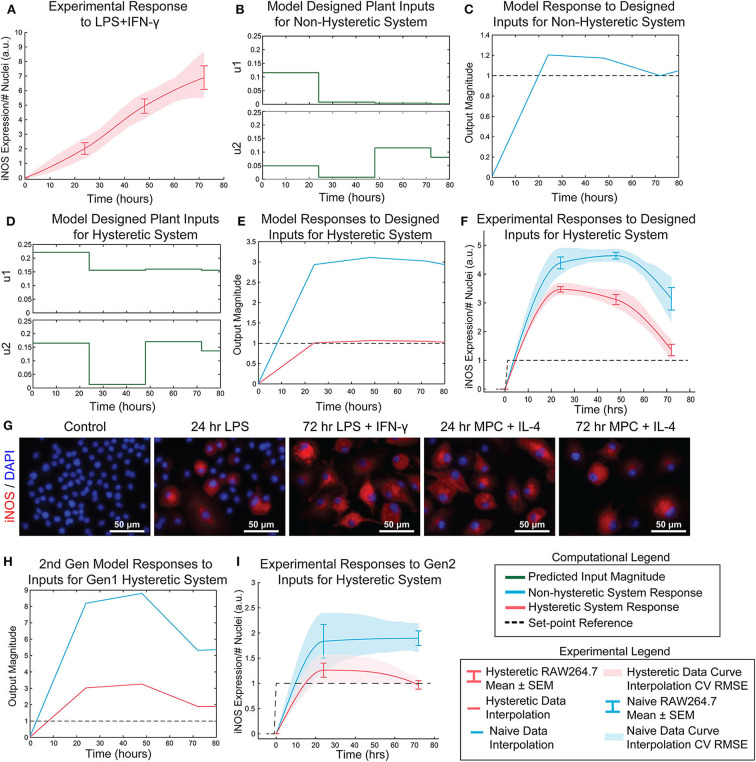
Open-loop control of pro-inflammatory macrophage activity is experimentally achieved using a nested multiple regression. **(A)** RAW 264.7 macrophage temporal response to 1 μg/mL LPS and 100 ng/mL IFN-γ. **(B)** Model designed inputs *u*_1_ and *u*_2_ using hysteresis-free model, which reflects cells beginning in a naïve state. **(C)** Hysteresis-free model response to inputs defined in **(B)**. **(D)** Model designed inputs *u*_1_ and *u*_2_ using first generation model accounting for hysteresis, which reflects cells starting from a non-naïve 24 h IL-4 primed state. **(E)** Hysteretic model (red) and non-hysteretic model (blue) responses to inputs defined in **(D)**. **(F)** Experimental delivery of designed inputs in **(D)** reflects predicted control output **(E)** for both hysteretic IL-4 primed (red curve, mean ± SEM, *N* = 16; interpolated curve ± RMS CV error) and non-hysteretic (blue curve, mean ± SEM, *N* = 16; interpolated curve ± RMS CV error) RAW 264.7 macrophage cultures. **(G)** Representative images of iNOS staining in model predictive control experiments using the inputs in **(D)**. **(H)** Simulation of updated 2nd generation model with dynamic supra-additivity term in response to designed inputs **(D)** captures experimental RAW 264.7 iNOS expression for both hysteretic (red curve) and non-hysteretic (blue curve) systems. **(I)** Experimental validation of the second-generation global model. Delivery of inputs designed to maintain a constant unit output of iNOS in a hysteretic system using the new model (inputs shown in Figure S6) improves control output for both hysteretic IL-4 primed (red curve, mean ± SEM, *N* = 8; interpolated curve ± RMS CV error) and non-hysteretic (blue curve, mean ± SEM, *N* = 8; interpolated curve ± RMS CV error) macrophage cultures.

Transfer functions were linearly combined with coefficients for supra-additivity (λ) and hysteresis (γ) acting as pre-processing filters, i.e., the terms were multiplied with each model's output, then added. The global regression of the function has the final form in Materials and Methods, Equation 10 [*R*^2^ = 0.748; *p*-value (vs. constant model) = 1.34e-38]. Simultaneous administration of unit, high, inputs *in vitro* vastly overshot the unit value of iNOS and did not settle over the course of the experiment (Figure 6A), demonstrating that it is possible to obtain sustained iNOS response, but that more carefully crafted input sequences are needed to obtain constant, sustained expression of iNOS. We therefore next used the global model (Figure 5F) together with an MPC controller to design input trajectories for LPS (u_1_) and IFN-γ (u_2_) needed to obtain sustained constant iNOS expression over a 72 h control horizon (Figure 6B). Using these trajectories, the simulated plant reached the reference value by 24 h with a minor overshoot that settled by 72 h (Figure 6C). Including hysteresis in the plant controller estimation increases the predicted inputs magnitude needed to obtain the unit step reference (Figure 6D). Given the input sequence defined in Figure 6D, a hysteretic system was predicted to respond with relatively small overshoot and error (Figure 6E, red curve). Importantly, the model captures the large overshoot that would be expected from administering elevated input levels to a non-hysteretic system (Figure 6E, blue curve).

Next, the relative input magnitudes defined for a hysteretic plant (Figure 6D) were translated to concentrations of LPS and IFN-γ, which were administered as temporally defined to RAW 264.7 macrophages in culture. The macrophage iNOS expression trajectories reflected the model predicted response for both hysteretic, i.e., pretreatment with 100 ng/mL IL-4 (Figure 6F, red curve and Figure 6G) and non-hysteretic (Figure 6F, blue curve) cell conditions. Since this initial model only accounted for a static supra-additivity term, we next updated it to incorporate a dynamic supra-additivity λ term that updated with time based on our response data in Figure 5A. The updated model was simulated with inputs used experimentally (Figure 6F) and defined by the original model (Figure 6D). This 2nd generation model improved the predictive performance with results that recapitulated the overshoot seen in the hysteretic system (Figure 6H). Since we wanted to ultimately achieve a unit reference system response, our last step was to use the 2nd generation model to define new system inputs (Supplementary Figure S6) for the IL-4 pre-treated hysteretic system using the MPC controller. We then applied these temporal input sequences to both blank media and IL-4 pre-treated macrophages. Excitingly, this MPC designed input sequence improved macrophage iNOS expression dynamic response because the IL-4 pre-treated cells settled to the target reference with minimal overshoot (Figure 6I). We also found that non-hysteretic (blank media pre-treated) cells overshot and did not settle to the reference by the control horizon (Figure 6I), as predicted by the model (Figure 6E).

In total, these experimental findings show that our global plant model predicts the dynamics macrophage pro-inflammatory response, including transient response to LPS, supra-additivity, and hysteresis. Moreover, we showed that this model could be used to define dual stimulation strategies that could prolong RAW 264.7 cell polarization as quantified by iNOS.

## Discussion

In this work, we developed a novel paradigm for engineering immune activity by defining predictive data-driven models of macrophage polarization and using them to define the dynamic delivery of pro-inflammatory factors to control the duration and magnitude of macrophage polarization. Rather than identifying detailed, highly parameterized mechanistic models, we applied a control theory framework to globally describe the pro-inflammatory activity of macrophages over time. Specifically, using expression of the canonical pro-inflammatory (M1) marker iNOS as an output, we defined a black-box transfer function to capture the dynamic response of macrophages given a temporal sequence of applied LPS and IFN-γ as system inputs. Our overall modeling framework coupled linear ARX models, which are uniquely identifiable, with non-linear elements that accounted for state-history dependent hysteresis and supra-additivity from multiple pro-inflammatory stimuli. Our global plant model structure not only predicted responses to different input sequences, but enabled design of new stimulation sequences that yielded a desired temporal iNOS response overcoming macrophage refractory behavior (Figure 6).

Immune dysregulation plays a central role in diverse diseases. Dysregulated activity of macrophages in particular can both hinder tissue repair and promote disease pathogenesis. However, macrophage functional diversity and broad distribution throughout the body also makes them excellent targets for modulating immune function to treat an array of diseases (Salim et al., 2016). Yet the vast majority of new immunomodulatory strategies, including inflammatory inhibitors and cell-based therapies, do not explicitly account for the temporal evolution of macrophage response needed to resolve the response to injury.

The importance of a temporally dynamic immune response has been highlighted by recent findings that long term resolution of inflammation depends on a sufficiently pro-inflammatory initial response followed by anti-inflammatory and resolving activity. Early pro-inflammatory macrophage response enables clearance of pathogens and damaged cells and subsequently triggers the anti-inflammatory and pro-regenerative response (Lee et al., 2016; Spiller and Koh, 2017; Ponzoni et al., 2018). Thus, in the current study, we sought to model and control macrophage pro-inflammatory activity, measured by iNOS expression. Using an ARX model structure, which is widely used for black-box system identification in engineering (Rachad et al., 2015) and biological systems (Liu and Allen, 2002; Zurakowski and Teel, 2006; Shin et al., 2012; Deshpande et al., 2014), we identified computational models able to predict and control temporal iNOS expression. This black-box approach enabled us to fit three parameters to model the dynamic LPS response and three more to fit the IFN-γ response, in contrast to dozens required in mechanistic differential equation models of macrophage polarization (Salim et al., 2016). A key feature of our black box modeling framework is that it is generalizable to broad inputs, outputs, and disease cases. Indeed, relationships between inputs and macrophage responses are quantitatively linked by experimental data, which can be extended beyond iNOS, LPS, and IFN-γ. This framework is therefore generalizable to inputs and outputs relevant to other diseases and markers of macrophage activity by experimentally tuning the model parameters to the new system.

Interestingly, when implementing model-predicted LPS input sequences, we observed that the time-dependent decay in the efficacy of LPS persisted. In fact, when the designed input magnitude was multiplied against a time-dependent decay term (Figures 3J,L, dashed lines), we were able to simulate the observed experimental response. This finding is consistent with macrophage auto-regulatory processes that prevent runaway inflammatory activity to LPS (Ziegler-Heitbrock et al., 1994).

The current work has some limitations that invite the need for future studies. First, we used murine RAW 264.7 immortalized macrophages, which is considered one of the best macrophage cell lines, for development of the methodology in this study, due to their high reproducibility between labs and studies (Taciak et al., 2018; Kong et al., 2019), but future work is needed to validate and tune the models for primary isolated macrophages. Further, to extend the utility of the model for disease therapeutics, it will be necessary to identify similarities and differences between primary macrophages, either bone-marrow derived or peritoneal, collected from wild type mice and mouse models of chronic inflammatory diseases. For example, macrophages are known to exhibit distinct inflammatory profiles from diabetic patients than from healthy individuals (Li et al., 2019), which will be reflected in the identified model parameters. Additionally, the methodology developed here lays a foundation for dynamic control of macrophage activation using a single polarization marker, but a wider panel of pro- and anti-inflammatory markers are needed to fully delineate macrophage activation state and effector function. Ultimately, the use of this methodology in *in vivo* models will be necessary to determine if it is possible to control immune activity for translational applications.

Together, our dynamic experimental and computational approach establishes a new way of conceptualizing and modulating macrophage activity by using a temporal sequence of input stimuli to shape the trajectory of inflammatory response. We experimentally validated the computational model predictions, extending previous theoretical work in model predictive control for patient-specific therapeutics (Day et al., 2010). We envision this framework having broad-reaching applications both *in vitro* an *in vivo*. Moreover, our ability to modulate macrophage activity suggests that design of temporally varying inputs has therapeutic potential for broad chronic inflammatory disorders.

## Materials and Methods

### RAW 264.7 Macrophage Cell Culture and Conditioning

All studies in this work were performed using RAW 264.7 murine immortalized macrophages (ATCC TIB-71™). Macrophages were expanded, maintained, and cultured in basal macrophage medium, which is comprised of DMEM (Thermo Fisher Scientific; 12430062), 10% FBS (Thermo Fisher Scientific; 26140079), and 1% antibiotic/antimycotic (Sigma-Aldrich; A5955). Cells were cultured to 70% confluence before conditioning began. Cells were conditioned by addition of medium with lipopolysaccharide (LPS; Sigma-Aldrich; L2880 and Invitrogen; 00-4976-93), interferon gamma (IFN-γ; R&D Systems; 485-MI), or interleukin (IL)-4 (PeproTech; 214-14) as indicated. RAW 264.7 macrophages were conditioned with LPS or IFN-γ alone to quantify individual stimulus dynamic response, with LPS or IFN-γ sequentially to recover iNOS expression via orthogonal input, or with LPS or IFN-γ simultaneously to quantify supra-additivity and model predictive control strategy response. Pre-treatment, 24 h of 100 ng/mL IL-4 prior to addition of LPS or IFN-γ, was used to induce an anti-inflammatory, non-naïve state for experiments involving hysteretic effects.

### Quantification of iNOS Expression via Immunofluorescence and Western Blot

For immunocytochemistry (ICC) experiments, macrophages were cultured in 96-well microplates. Macrophages were fixed in 4% PFA solution for 15 min and blocked with 5% BSA + 3% goat serum in PBS for 1 h. Cells were stained with α-iNOS antibody (Cell Signaling Technology; Cat. No. 13120; 1:400) and DAPI for normalization to nuclei count. Cells were imaged at 10X magnification (Zeiss Observer Z1). Image fluorescence was thresholded and total fluorescence above the threshold was normalized to nuclei number.

For Western blot experiments, cells were cultured in 6-well plates then lysed using RIPA buffer with PMSF (Sigma-Aldrich), and cOmplete Mini (Sigma-Aldrich). Membranes were probed for α-tubulin (Sigma-Aldrich, Cat. No. T6074; 1:4,000) and iNOS (1:1,000). Membranes were imaged on a LiCor Odyssey CLx machine and quantified in ImageStudio Lite. iNOS band intensity was normalized to α-tubulin intensity to yield iNOS expression.

### Data Normalization and Dynamic iNOS Response Figure Generation

ICC and Western blot data were aggregated and iNOS expression for each independent experiment was normalized to the positive control with RAW 264.7 cells treated with 1 μg/mL LPS for 24 h. iNOS dynamics plots were generated using the Gramm package for MATLAB (Morel, 2018). Data at sampled time points (0, 24 48, and 72 h) were expressed as mean ± SEM for separated data (*N* = 38 for LPS single input experiments; *N* = 8 for LPS repeated input experiments; *N* = 8 for LPS cycled input experiments; *N* = 32 for IFN-γ single input experiments; *N* = 16 for IFN-γ repeated input experiments; *N* = 16 for IFN-γ cycled input experiments per each time point. Sample sizes used for model fits are indicated in figure legends). To generate interpolation curves, data were smoothed using the Savitzky-Golay (sgolay) option in the curve fitting toolbox. Shaded band on curve represents root mean squared (RMS) cross validation error on smoothed data (Morel, 2018).

### SISO and MISO Linear ARX Model System Identification

LPS response data were compiled into a time-domain data object with experiments for all input concentrations and unique input sequences. Dynamic models were fit (Supplementary Table S1) to the autoregressive with exogenous inputs (ARX) model structure

(1) $$\begin{array}{r} A\left(z^{-1}\right)y\left(t\right)=\;B\left(z^{-1}\right)u\left(t-n_{k}\right)+\;\varepsilon \left(t\right) \end{array}$$

where u(t) is the LPS stimulation input, *n*_*k*_ is the system dead time, y(t) is the iNOS response, and the model coefficients consist of

(2) $$\begin{array}{r} A\left(z^{-1}\right)=1+\;a_{1}z^{-1}\;+\;a_{2}z^{-2}+\;\ldots \;+\;a_{n}z^{-n_{a}} \end{array}$$

(3) $$\begin{array}{r} B\left(z^{-1}\right)=\;b_{0}\;+\;b_{1}z^{-1}+\;b_{2}z^{-2}+\;\ldots \;+\;b_{n_{b}}\;z^{{-n}_{b}} \end{array}$$

with one pole (n_a_), two zeros (n_b_), an input-output delay of 1 time step corresponding to 24 h, and zero initial conditions (System Identification toolbox, MATLAB, 2018b). Parameters were estimated by solving the least squares problem

(4) $$\left(W^{\text{T}}W\right)\theta =W^{T}y_{m}$$

where **W** is the 4 × 4 regressor matrix consisting of given inputs, $y_{m}={\left[y\left(0\right)\;y\left(1\right)\;y\left(2\right)\;y\left(3\right)\right]}^{T}$ is the measured output vector, and the uniquely identified solution to the least squares parameter estimation is

(5) $$\theta ={\left[a_{1}\;a_{2}\;\ldots \;a_{n_{a}}\;b_{0}\;b_{1}\;\ldots b_{n_{b}}\right]}^{T}$$

The sampling time step of the identified model was set to 24 h, which was equal to the data acquisition time step.

Realized for control design and flow diagram integration, the canonical state space equations for this ARX model are of the form Equations (6) and (7) with matrix coefficients listed in Supplementary Table S2.

(6) x(t+1)=Ax(t)+Bu(t)

(7) y(t)=Cx(t)+Du(t)

where **A** is the 2 × 2 system matrix, **B** is the 2 × 1 input matrix, **C** is the 1 × 2 output matrix, **D** is the 1 × 1 feedthrough matrix, and *t* is discrete time. Model order was selected to minimize the small sample-size corrected Akaike's Information Criterion (AICc) (Ljung, 1999) and mean squared error (Supplementary Table S3). This process was repeated for a SISO IFN-γ model (n_a_ = 1, n_b_ = 2) and a multi-input single output (MISO) model with both LPS and IFN-γ inputs (n_a_ = 1, n_b_ = 2 for both inputs).

### LPS System Controller Design

Controller design was carried out in the Control System Designer application (MATLAB, Mathworks) to find an input strategy capable of achieving the unit step response from a step reference. Since our estimated system dynamics indicated a continuous time zero at the origin, we selected a PI controller to compensate because it adds a continuous time pole at the origin and is widely used in engineered systems (Nise, 2015). A proportional-integral (PI) controller (Equation 8; Bellman, 1961), was designed with robust noise and quick response specifications (parameters given in Supplementary Table S4). In discrete time, the PI control law specifies the input in the current time step as a function of the current and prior errors (Ogata, 1995; Nise, 2015):

(8) $$u\left(\text{t}\right)={\text{K}}_{\text{p}}e\left(\text{t-1}\right)+{\text{K}}_{i}\sum_{0}^{\text{t-1}}e\left(\bar{\text{t}}\right)$$

where K_p_ is the proportional gain associated with the error in the last time step and K_*i*_ is integral gain associated with the sum of errors in the prior time step. Additionally, since our system model (Equation 1), enabled state estimation, we implemented a third order linear-quadratic Gaussian (LQG) controller, defined to minimize $\tilde{J}$

(9) $$\tilde{J}=\sum_{\text{t=0}}^{\text{N-1}}\left(x{\left(t\right)}^{T}Qx(t)+u{\left(t\right)}^{T}Ru(t)\right)+x{\left(N\right)}^{T}Q_{F}x(N)$$

The controller was tuned to be robust to noise and assuming moderate measurement noise (zero/pole/gain parameters in Supplementary Table S4). Where N is the time horizon, *t* is the time step, Q is the state cost matrix, Q_f_ is the final state cost matrix, and R is the input cost matrix. Q, Q_f_, and R were defined internally by the system designer application.

### Surface Interpolation for Non-linear Model Elements Parameterization

#### Supra-Additive Pro-Inflammatory Surface

Data matrices across concentration gradients of simultaneous LPS and IFN-γ addition were divided by the iNOS expression level given LPS only for each concentration to give the ratio by which each IFN-γ concentration amplified iNOS expression. The discrete matrix data were fit using cubic interpolation (Curve Fitting Toolbox) for each sampled time point. The cubic interpolation minimized the root mean square error between the fitted and actual values while avoiding outliers from overfitting for the supra-additivity matrix (Supplementary Figure S7; data used for interpolation are provided in Supplementary Data 1). Other curve fits sampled were linear interpolation, polynomial models, spline interpolation, and local linear regression (Lowess) but had greater error and were subject to overfitting. The resulting scaling factor, λ, was queried for intermediary concentrations of each input at each sampled time.

#### M2 Hysteresis Surface

For each LPS concentration, iNOS expression for non-M2 polarized LPS-only treated cells were divided by iNOS expression values from cells treated with an array of IL-4 concentrations for 24 h followed by 24 h of LPS. The matrix of LPS and IL-4 concentrations was interpolated using 3rd order polynomial linear regression, where parameters (Supplementary Table S6) were estimated by the least-squares method, which provided inverse of the continuous input concentration- dependent attenuation factor γ. Other models were assessed as above, considering overfitting via leave N out cross validation (with 10% of samples left out) and root mean square error minimization (Supplementary Figure S7).

### Global System Model Architecture and Formulation

For our first nested model, we used a multiple regression with interaction terms to quantify the supra-additive effect of adding both IFN-γ and LPS. Simulations were run using SISO models for single- and double- stimuli experimental results to populate a table with predicted output levels for varying magnitudes of input. The linear dual-input (both IFN-γ and LPS for all time points) model predictions were used as the regression output *y*, and the single input (either IFN-γ or LPS) SISO model predictions were given as regression inputs to fit a model of relative contributions of time and input interactions ($y_{LPS}^{\prime }$ and $y_{IFN\gamma }^{\prime }$). The terms that significantly predicted total iNOS output *y* were time-dependent LPS concentration, time-dependent IFN-γ concentration, and a combinatorial effect of both LPS and IFN-γ inputs (Equation 10). Weighting coefficients, *c*, for each term are given in Supplementary Table S5.

(10) $$\begin{array}{r} y=\left[c_{1}ty_{LPS}^{\prime }\;+\;c_{2}ty_{IFN\gamma }^{\prime }\right]\;+\;c_{3}y_{LPS}^{\prime }y_{IFN\gamma }^{\prime } \end{array}$$

We next sought to construct a second global model structure that handles time- and concentration-dependent supra-additive interaction terms. Here, experimentally obtained iNOS expression data given varying concentrations of LPS and IFN-γ was fit to a response surface, as described above, for each time point. This surface was used to define a table as above but with time and input-dependent dual-input model output predictions. A multiple linear regression on this prediction table similarly fit coefficients for time and input interaction terms (Equation 10, Supplementary Table S5). We accounted for this temporally shifting interaction term by implementing the multiple linear regression model with the output from the identified SISO transfer function models *and* time as inputs and the MISO transfer function output as multiple regression model output,

### Global System Model MPC Controller Design and Prediction

The Model Predictive Control toolbox in MATLAB (2018b) was used to create the controller and define manipulated input sequences for the MISO “global” model. The SISO IFN-γ and LPS transfer functions with weighting coefficients derived from the multiple regression was given as the model object, referred to as the plant (Equation 11, Figure 1B). The plant model was defined with two manipulated variable inputs, one output, a control horizon of 72 h, and a prediction horizon of 120 h. Manipulated variables were constrained with a minimum of 0, a maximum of 1, and unconstrained rates of change. The default state estimator (Kalman filter) settings were used for the controller predictions (MATLAB). Closed loop simulations generated the inputs, *u*, needed to obtain the set reference (unit step) over simulation time with the expected system output *y*. Plant performance was evaluated by running open-loop simulations given the predicted inputs from the closed-loop simulation. Optimal predicted input and output trajectories were validated using the mpcmove function.

(11) $$\begin{array}{r} \text{G}=\left[{\text{C}}_{1}{\text{Y}}_{1},\;{\text{C}}_{2}{\text{Y}}_{2}\right]+{\text{C}}_{3}{\text{Y}}_{1}{\text{Y}}_{2} \end{array}$$

## Data Availability Statement

The datasets generated for this study are available on request to the corresponding author.

## Author Contributions

LDW and LBW designed the study. LDW, JF, and AW conducted experiments and data analysis. LDW conducted computational predictive modeling. JU conceptualized physical interpretation of control laws. LBW supervised the project. All authors contributed to the article and approved the submitted version.

## Conflict of Interest

The authors declare that the research was conducted in the absence of any commercial or financial relationships that could be construed as a potential conflict of interest.
