# Supplementary material for: Experimental Control of Macrophage Pro-Inflammatory Dynamics Using Predictive Models
Source: Front Bioeng Biotechnol. 2020 Jul 13;8:666. doi: 10.3389/fbioe.2020.00666 (PMC7381235; doi:10.3389/fbioe.2020.00666)
Supplement: Supplementary file 1 [file Data_Sheet_1.pdf]

Supplementary Material

Experimental control of macrophage pro-inflammatory dynamics using predictive models

Weinstock *et al.*

1 Supplementary Figures

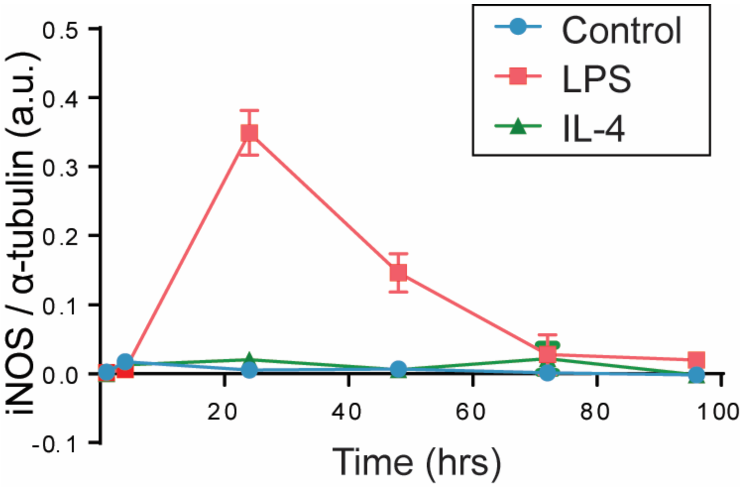

Supplementary Figure S1. Western blot quantification of *in vitro* RAW264.7 macrophage iNOS protein expression after treatment with LPS, IL-4, or control media shows iNOS peaks at 24 hrs of LPS treatment but is not expressed in IL-4 conditions (n=2; mean±min/max range).

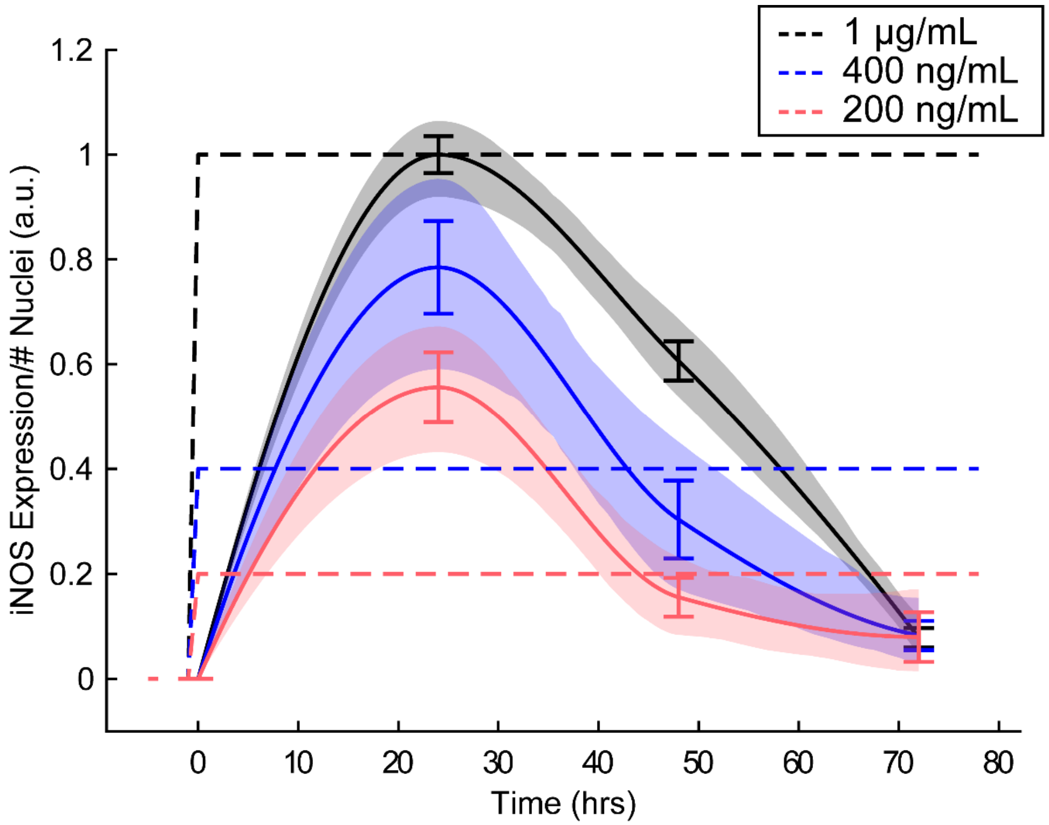

Supplementary Figure S2. Temporal dynamics of iNOS expression are conserved across LPS concentrations and magnitude of response is monotonic. Dashed lines represent relative LPS input. iNOS expression are mean±SEM, N=24, 40, and 16 for 200 ng/mL; 24, 24, and 16 for 400 ng/mL; 32, 40, and 16 for 1 μg/mL at 24 hours, 48 hours, and 72 hours, respectively. Solid curves were interpolated with shaded region showing RMS CV error.

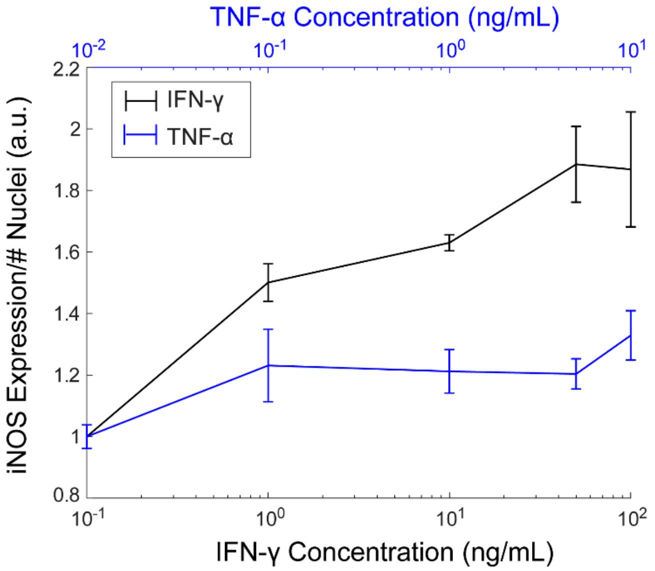

Supplementary Figure S3. Choice of orthogonal pro-inflammatory input. RAW 264.7 iNOS expression after 24 h treatment with a range of TNF-α (blue) or IFN-γ (black) concentrations.

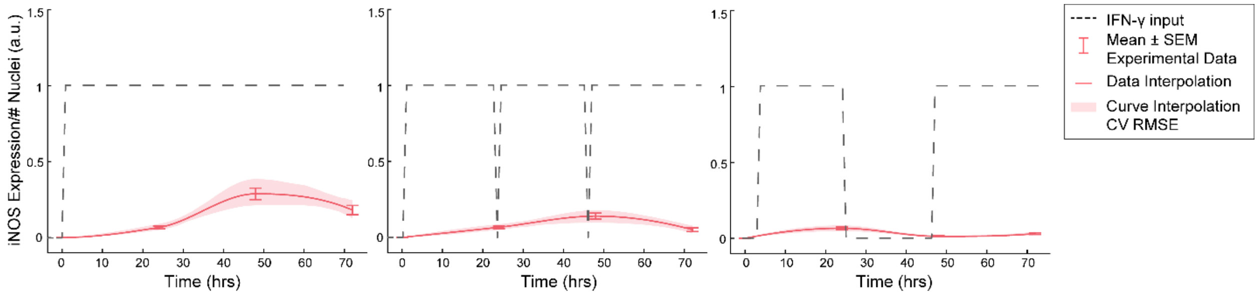

Supplementary Figure S4. RAW 264.7 macrophage temporal dynamic response to 100ng/mL IFN-γ alone is distinct from the LPS response but is also not sustained.

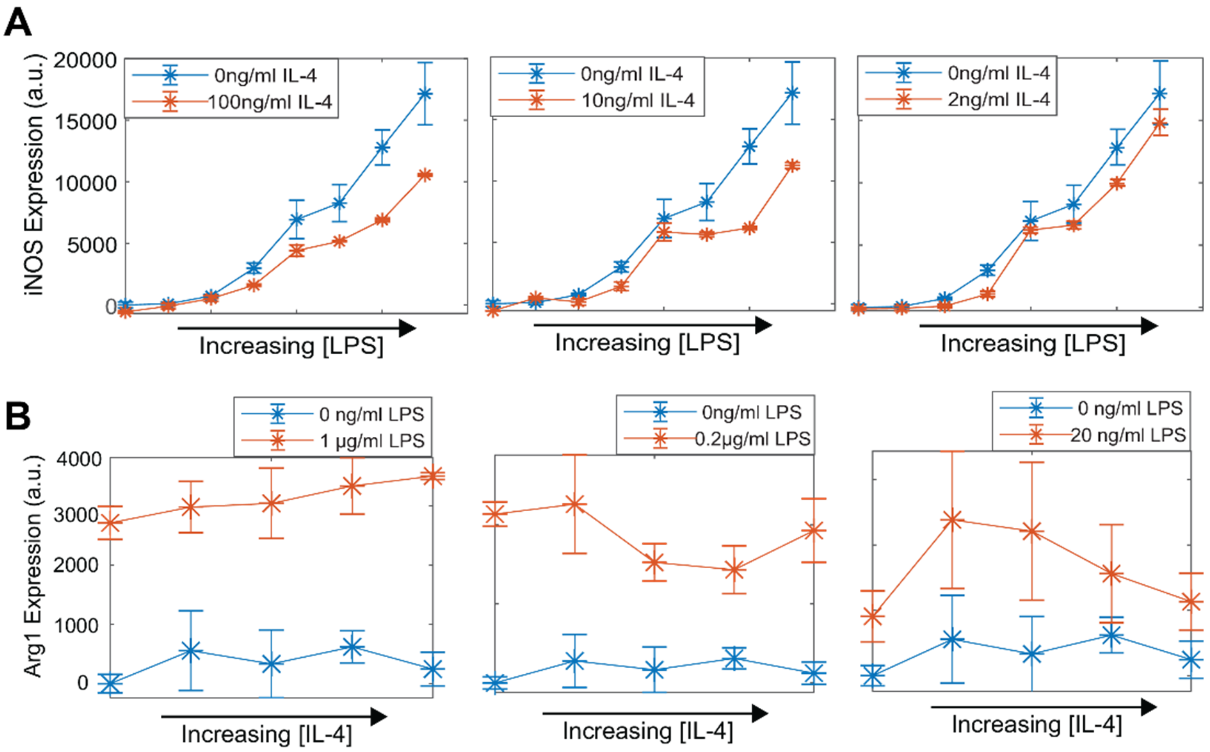

Supplementary Figure S5. Prior M1 state polarization increases, rather than decreases, subsequent M2 polarization. Macrophages were treated with a range of LPS concentration for 24 hrs to induce initial M1 polarization. Subsequently, a range of IL-4 doses were added for all LPS concentrations. (**A**) Increasing IL-4 concentrations attenuated iNOS expression for high LPS concentrations. (**B**) Addition of LPS stimulates Arg1 expression compared to conditioning with 24 hrs of IL-4 alone, showing a primed polarization toward M2 given prior M1 activation.

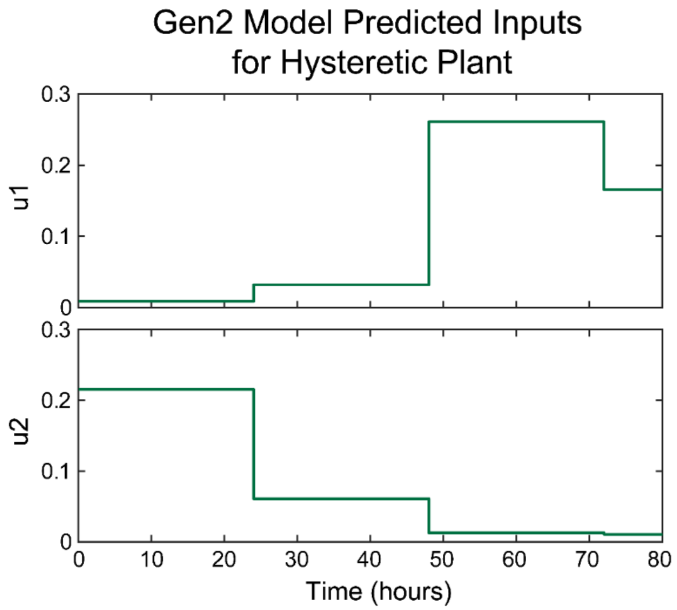

Supplementary Figure S6. Second generation model predicts a temporal input sequence to control hysteretic RAW264.7 macrophages. Controller designed inputs u_1 (LPS) and u_2 (IFN-γ) for the second-generation model to control a hysteretic plant. Experimental implementation of these inputs generated results shown in **Figure 6I**.

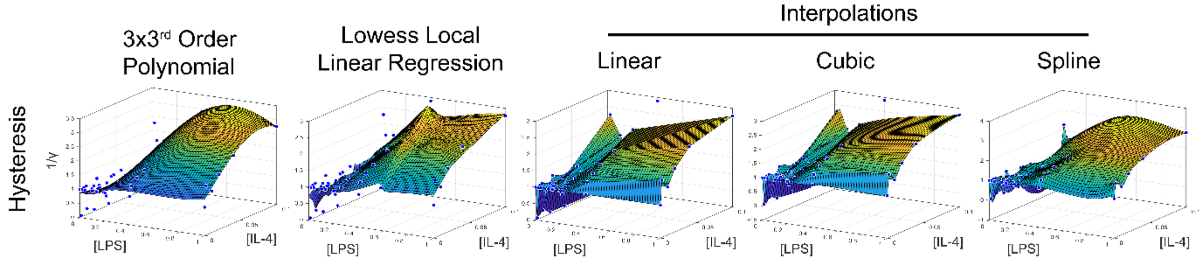

RMSE:	0.3843	0.3578	0.5882	0.5819	0.6723
Constraint Violation:	No	No	No	No	No
Smooth Topology:	Yes	No	Yes	Yes	No

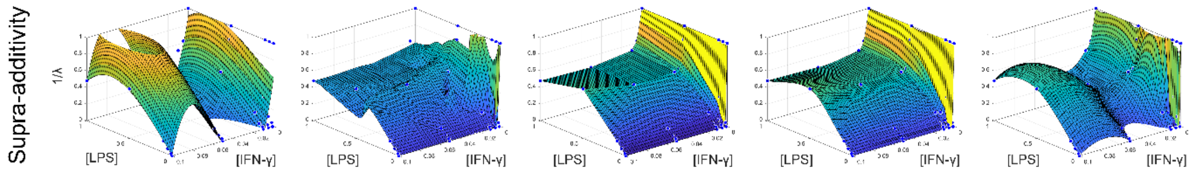

RMSE:	0.2683	0.2089	0.4581	0.4459	0.3044
Constraint Violation:	Yes	Yes	No	No	Yes
Smooth Topology:	Yes	No	Yes	Yes	Yes

Supplementary Figure S7. Selection of surface models for inverse gamma and lambda elements. Hysteresis and supra-additivity surfaces were generated using several models. The models selected for use were the 3-by-3rd order polynomial and cubic interpolation fits, respectively. The supra-additivity plots shown are for the 24 hr data, and 48 and 72 hr data were fit using the same cubic interpolation method. Models were assessed by minimizing root mean square error (RMSE), constraint violation, and topology quality across 100 iterations of leave-N-out-cross-validation where N was 10% of data. Constraints were violated if the surface models predicted values below $0 < 1/\gamma$ for hysteresis or $0 < 1/\lambda < 1$ for supra-additivity. Surface quality, or smoothness, indicates no surface buckling or folding, which would cause large output changes given small changes in inputs.

2 Supplementary Figures Tables

Supplementary Table S1. ARX polynomial coefficients.

		z^0	z^{-1}	z^{-2}
**LPS**	A	1	-0.3163	---
	B	0	0.81	-0.7727
**IFN-γ**	A	1	-0.3849	---
	B	0	0.0634	0.0566
**LPS + IFN-γ**	A	1	-0.76	---
	B1	0	0	1.252
	B2	0	2.019	0

Supplementary Table S2. LPS transfer function coefficients.

		LPS Model	IFN-γ Model	LPS + IFN-γ Model
**A**	A_{11}	0.3163	0.3849	0
	A_{12}	0	0	0
	A_{21}	0.5	0.5	1
	A_{22}	0	0	0.76
**B**	B_{11}	2	0.5	0.6262
	B_{12}	---	---	0
	B_{21}	0	0	0
	B_{22}	---	---	1.009
**C**	C_1	0.405	0.1268	0

	C_2	-0.7727	0.2263	2
**D**	D_1	0	0	0
	D_2	---	---	0

Supplementary Table S3. LPS ARX model AICc and MSE for parameter number, n_a and n_b, ranging from 1-4.

		**n_a**			
	**n_b**	1	2	3	4
**AICc**	1	331.62	430.59	548.77	707.25
	2	425.95	383.23	561.34	697.86
	3	550.49	562.75	574.56	711.95
	4	640.84	683.44	697.82	1789.39
**MSE**	1	0.10	0.01	0.12	0.22
	2	0.04	0.01	0.12	0.21
	3	0.14	0.13	0.13	0.21
	4	0.17	0.20	0.20	0.21

Supplementary Table S4. PI controller parameters and LQG controller design.

**PI Controller Parameters**			**LQG Controller Parameters**		
**K_p**	**K_i**	**T_s**	**Zeros**	**Poles**	**Gain**
0.401	0.0334	24	-2.631	1.0	0.1966
			0.4089	0.9539	

Supplementary Table S5. Multiple regression interaction terms, coefficients, and p-values.

Term	Estimate	p-value
**Time : LPS-induced iNOS**	0.1696	1.299e-08
**Time : IFN-γ-induced iNOS**	0.3458	6.485e-07
**LPS-induced iNOS : IFN-γ-induced iNOS**	69.738	7.366e-11

Supplementary Table S6. Parameters for 3-by-3rd order polynomial hysteresis surface model of form: $p_{00} + p_{10}*x + p_{01}*y + p_{20}*x^2 + p_{11}*x*y + p_{02}*y^2 + p_{30}*x^3 + p_{21}*x^2*y + p_{12}*x*y^2 + p_{03}*y^3$.

n	0	1	2	3
p_{0n}	0.797	11.65	280	-864.8
p_{1n}	1.643	58.16	-431	
p_{2n}	0.4276	-7.363		
p_{3n}	-0.7914			

3 Supplementary Data Caption

Supplementary Data 1. RAW264.7 macrophage iNOS expression data for 24, 48, and 72 hour time points conditioned with 0 to 1 µg/mL and 0 to 100 ng/mL. Data (average of N=2 samples) are also represented in the heatmaps of **Figure 5A**. Data are used in surface fitting by cubic interpolation to obtain the global, MISO model supra-additivity (λ) term. Data are normalized by 24 hour 1 µg/mL LPS positive control values on each plate for all time points.
